# One-step selection of Vaccinia virus-binding DNA aptamers by MonoLEX

**DOI:** 10.1186/1472-6750-7-48

**Published:** 2007-08-15

**Authors:** Andreas Nitsche, Andreas Kurth, Anna Dunkhorst, Oliver Pänke, Hendrik Sielaff, Wolfgang Junge, Doreen Muth, Frieder Scheller, Walter Stöcklein, Claudia Dahmen, Georg Pauli, Andreas Kage

**Affiliations:** 1 Centre for Biological Safety 1, Robert Koch-Institut, Nordufer 20, 13353 Berlin, Germany; 2 Department of Biology/Chemistry, Division of Biophysics, University of Osnabrueck, 49069 Osnabrueck, Germany; 3 Biosystems Technology, University of Applied Sciences Wildau, Bahnhofstr. 1, 15747 Wildau, Germany; 4 Institute of Biochemistry and Biology, Department of Analytical Biochemistry, University of Potsdam, Karl-Liebknecht-Str. 24-25, 14476 Golm, Germany; 5 AptaRes AG, Im Biotechnologiepark TGZ I, 14943 Luckenwalde, Germany; 6 Institute of Laboratory Medicine and Pathobiochemistry, Charité Universitätsmedizin Berlin, Westend Haus 31, Spandauer Damm 130, 14050 Berlin, Germany

## Abstract

**Background:**

As a new class of therapeutic and diagnostic reagents, more than fifteen years ago RNA and DNA aptamers were identified as binding molecules to numerous small compounds, proteins and rarely even to complete pathogen particles. Most aptamers were isolated from complex libraries of synthetic nucleic acids by a process termed SELEX based on several selection and amplification steps. Here we report the application of a new one-step selection method (MonoLEX) to acquire high-affinity DNA aptamers binding Vaccinia virus used as a model organism for complex target structures.

**Results:**

The selection against complete Vaccinia virus particles resulted in a 64-base DNA aptamer specifically binding to orthopoxviruses as validated by dot blot analysis, Surface Plasmon Resonance, Fluorescence Correlation Spectroscopy and real-time PCR, following an aptamer blotting assay. The same oligonucleotide showed the ability to inhibit *in vitro *infection of Vaccinia virus and other orthopoxviruses in a concentration-dependent manner.

**Conclusion:**

The MonoLEX method is a straightforward procedure as demonstrated here for the identification of a high-affinity DNA aptamer binding Vaccinia virus. MonoLEX comprises a single affinity chromatography step, followed by subsequent physical segmentation of the affinity resin and a single final PCR amplification step of bound aptamers. Therefore, this procedure improves the selection of high affinity aptamers by reducing the competition between aptamers of different affinities during the PCR step, indicating an advantage for the single-round MonoLEX method.

## Background

New intervention strategies are required to detect, prevent and control disease outbreaks. In this context, orthopoxviruses (OPV) like Variola virus, Monkeypox virus and bioengineered OPV are often discussed to be potentially used as biological weapons [1]. Therefore there is general consent that the establishment of a comprehensive pool of antiviral drugs, including substances that inhibit OPV replication by mechanisms other than the substances already characterized, is a reliable strategy for treating and preventing smallpox and other OPV infections in humans [2].

Aptamers promise to be such additional candidates for prophylaxis and treatment of infectious diseases, as they can be directed against a wide variety of target molecules like toxins or even complete microorganisms [3-7]. Aptamers are short DNA or RNA oligonucleotides and, like antibodies, bind to their target by their three-dimensional structure with high affinity and specificity. Compared to antibodies, aptamers have several advantages, as they are selected *in vitro *which also enables selection against toxic or weakly immunogenic targets. DNA aptamers are heat and protease resistant without stabilizing modifications. Due to chemical synthesis, aptamer production can easily be scaled up [3]. During the selection process aptamers with the highest affinity to a target structure are isolated from a pool of oligonucleotides with random positions for nucleic acids, which, depending on the length of the aptamers, contain up to 10^15 ^different sequence variants, representing a significantly larger pool of variants than antibodies do. In addition to these evident benefits, aptamers exhibit some drawbacks, including their reduced resistance to nucleases and the problem of delivery and clearance in therapeutic application. First attempts to stabilize aptamers were promising [8,9].

The enrichment procedure that has commonly been used after being first reported in 1990 was called Systematic Evolution of Ligands by Exponential Enrichment (SELEX) [10,11]. Since then, great efforts have been made to improve this method [12,13], and many different aptamers have been selected by various SELEX-based protocols for a wide variety of targets ranging from small molecules to whole cells [14] and bacteria [15]. Some of these aptamers were shown to possess inhibitory activity with certain HIV strains [16], to block cell binding of human cytomegalovirus (CMV) [17] or influenza virus hemagglutinin [18] or were selected as tools for differentiation of closely related influenza strains [19]. Recently, the first aptamer-based drug Macugen was approved by the FDA for treating wet forms of macula degeneration, which is an important step towards the acceptance of aptamers in clinical therapy [20]. Further drug candidates are under clinical trials [21]. Finally, an aptamer-based detection device for cocaine already in practical application has been published recently [22].

**Figure 1:**
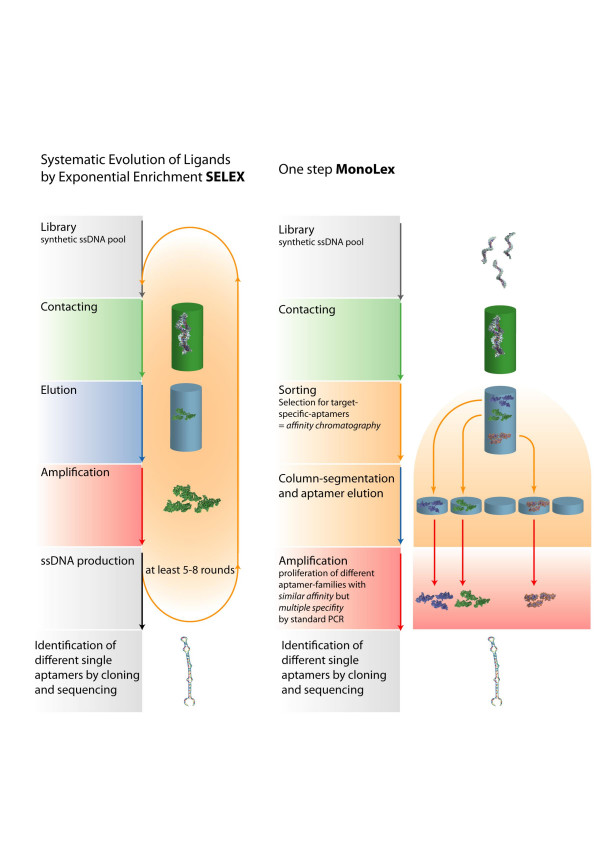
**Schematic presentation of MonoLEX in comparison to SELEX**. Based on a combinatory oligonucleotide library, SELEX comprises several cycles of target binding, elution and amplification of putative aptamers. In contrast, MonoLEX starts with one affinity chromatography to sort non-binding oligonucleotides, low-affinity aptamers and high-affinity aptamers. Highly affine aptamers are amplified once and characterized further by an aptamer blot assay.

As an alternative to the SELEX process with 7 to more than 30 selection and amplification cycles requiring large amounts of the target molecule, a new selection process was established. Based on reports about selecting functional oligonucleotides [23] and the potential of oligonucleotides as non-Watson-Crick-type binders to peptides and proteins [24], the present study applied this new one-step aptamer isolation protocol (MonoLEX) to retrieve DNA aptamers that have the potential to bind specifically to OPV particles. The MonoLEX approach combined a single affinity chromatography step with subsequent physical segmentation of the affinity resin and one single final exponential amplification step of bound aptamers. A schematic comparison of SELEX and MonoLEX is given in figure 1. Specific aptamers were selected from a combinatory library of oligonucleotides characterized by two flanking primers of known sequence and an internal region of 20 random nucleotide positions (N20 DNA library). Under adequate chromatographic conditions, like flow laminarity, sufficient capacity and homogeneity of the resin, high-affinity-binding aptamers stuck to the target, whereas weakly binding oligonucleotides could be removed from the resin by ample washing. Since the selection is an *in vitro *process, depending on the final application of the aptamer, the selection conditions can be adapted accordingly.

## Results

### Identification of high-affinity-binding aptamers by MonoLEX

**Figure 2:**
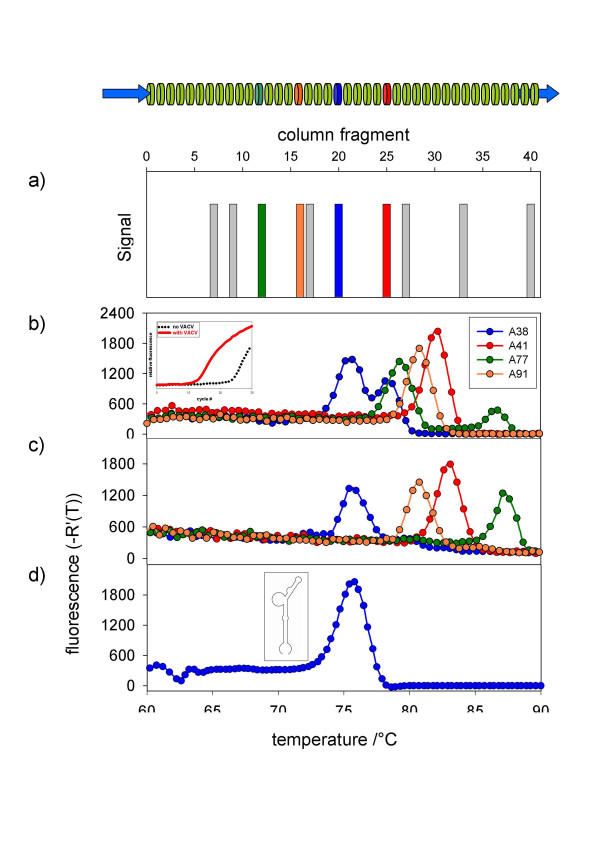
**Identification of high-affinity-binding aptamers by MonoLEX**. A combinatorial DNA library (64 b in length) was applied to an affinity capillary column coated with complete heat-inactivated VACV. Bound aptamers were desorbed from cut column slices and amplified by real-time PCR. (a) Data derived from different segments along the affinity column show a cumulation of aptamers in distinct segments while other segments do not amplify bound aptamers. Color labels indicate segments which were used for further evaluation of the aptamer pools. Figure 2b to 2d show the change of fluorescence per change of temperature plotted versus the temperature. (b) Melting temperature analysis of polyclonal aptamer pools after PCR amplification showing aptamer pools with different nucleic acid composition. In two of the pools (A38 and A77) more than one melting temperature maximum was observed, indicating different aptamer sub-pools. (c) Melting temperature analysis after aptamer dot blotting with the target molecule and repeated amplification. In A38 and A77 only one of the two sub-pools was amplified, indicating that one aptamer is more efficiently amplified after binding. The inset shows the preceding amplification results of an aptamer blotting assay with VACV (red solid line) and a negative control (black dotted line). The relative fluorescence is plotted vs. the cycle number. (d) Melting profile of the chemically synthesized and further characterized A38 with its predicted two-dimensional structure.

To select DNA aptamers specific for Vaccinia virus (VACV) as a model for OPV, aptamer selection was performed in two steps. An initial chromatography was performed on proteins from virus-free cell culture supernatant to eliminate aptamers binding to cell debris or media components. Subsequently, a second chromatography was carried out on resin-bound heat-inactivated VACV particles. For direct recovery of high-affinity-binding aptamers, the resin of the affinity column was physically segmented into slices and the amount of retained binding aptamers was estimated by quantitative real-time PCR. High concentrations of aptamers not eluted during the extended washing procedure were identified on several affinity column segments as indicated by the different grey and colored bars in Fig. 2a. Indicated column fragments contained aptamer amounts that significantly differed from the background. A subsequent fluorescence curve melting analysis (FCMA) of the PCR products revealed clearly distinguishable single or double melting peaks for four aptamers which are shown in color in Fig 2a. The different melting temperatures observed for these four aptamers indicate different nucleic acid composition of the oligonucleotides retained in distinct positions on the affinity resin (Fig. 2b). As observed for the two aptamers A38 and A77 (blue and green line in Fig 2a), more than one melting peak suggested the presence of at least two different aptamers with different melting temperatures amplified from one affinity column segment.

The sequences of these aptamers were determined by pyrosequencing as described above. None of the aptamers showed sequence homology to known viral or cellular sequences in the variable region as proven by BLAST search, neither including nor excluding the flanking primer sequences.

To verify the binding specificity of the identified aptamers, several methods were applied. First, an aptamer blotting assay was performed binding aptamers to heat-inactivated VACV. Bound aptamers were quantified using real-time PCR, revealing a significant enrichment of aptamer molecules as indicated by a C_T_-value shift of 10 (Fig. 2c, inset, red line) in relation to the background without VACV (Fig. 2c, inset, black dotted line). Additional peaks seen in FCMA directly after amplification of the aptamers 38 and 77 (A38 and A77, Fig. 2b) disappeared after the aptamer-blotting assay, resulting in one distinct peak for each aptamer (Fig. 2c). Interestingly, in this step the more prominent peak of A77 at 80°C vanished while the less prominent one at 87°C increased significantly in height. This finding implies that co-amplification of two aptamers can result in suppression of one aptamer. This effect is probably caused by significant differences in PCR efficiency when amplifying two aptamers of different composition, but is independent of the aptamer's affinity to the target.

Since A38 showed the highest antiviral activity to VACV in a preliminary screening, it was chosen for further characterization and binding studies. The A38 sequence is TACgACTCACTATAgggATCCTgTATATATTTTgCAACTAATTgAATTCCCTTTAgTgAgggTT. Figure 2d shows the melting profile of the chemically synthesized A38 as single peak; its two-dimensional structure under the reaction conditions used as obtained using the m-fold software [25] is presented in the inset.

### *In vitro*-binding studies of A38

**Figure 3:**
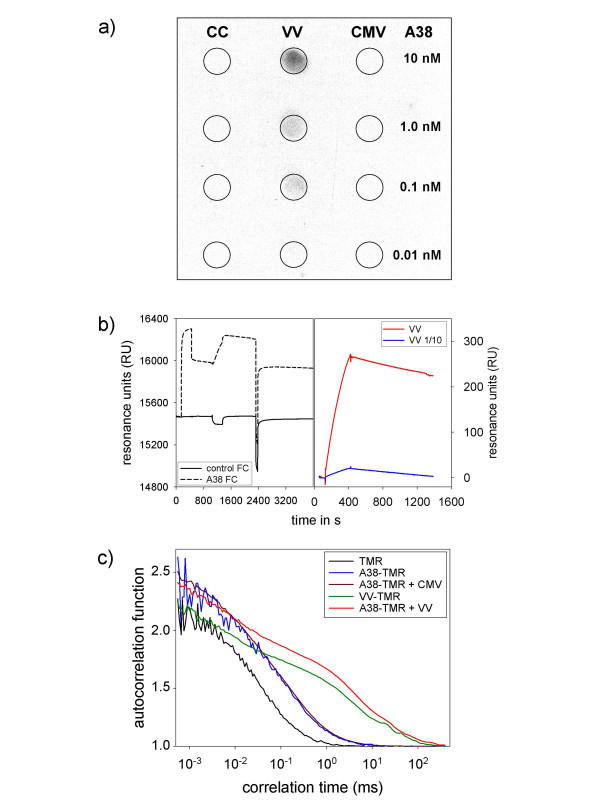
***In vitro*-binding studies of A38**. (a) Dot blot of VACV (VR-1536), non-infective cell culture supernatant (CC) and CMV, followed by incubation of biotin-conjugated A38 in various concentrations and labeling with a streptavidin peroxidase conjugate. (b) Surface Plasmon Resonance measurements (Biacore) of A38. Sequence of injections (injection start): A38 (150 s); VACV (1050 s); 50 mM sodium carbonate (2320 s); 1:10 diluted VACV (4080 s). The overlaid VACV net binding curves are shown on the right hand side. (c) Fluorescence Correlation Spectroscopy (FCS). Normalized autocorrelation functions (ACF) of tetramethylrhodamine isothiocyanate (TMR): free in solution (black), coupled to A38 alone (blue), coupled to A38 with added CMV (brown), coupled to A38 with added VACV (red), and directly coupled to VACV (green). Control experiments with CMV (brown) and 200-nm latex beads (not shown) mixed with TMR-conjugated A38 showed identical diffusion times to those for TMR-labeled A38 alone, indicating a binding exclusively to VACV particles.

Binding characteristics of A38 to VACV were further validated by dot blot analysis of immobilized VACV particles stained with biotinylated A38 (OligoService, Berlin, Germany). As shown in Fig. 3a, A38 binds exclusively to VACV particles and not to the non-infected corresponding cell culture supernatant or Cytomegalovirus (CMV) particles. Even in high concentrations of 10 nM aptamer, binding is still specific for VACV. The binding to VACV particles was concentration dependent and could no longer be observed for A38 concentrations below 10 pM.

As shown in Fig 3b, Surface Plasmon Resonance Spectroscopy (Biacore) confirmed a significant binding of VACV particles in suspension to immobilized A38. The binding was nearly linear for undiluted (red line) and diluted VACV suspensions (blue line). The slope was proportional to the virus concentration when the drifting baseline caused by aptamer leakage was considered. The linear slope indicates that the virus concentration was low (10^9 ^particles/mL for undiluted virus solution) and far from the saturation stage. A signal increase could be seen in spite of the extremely low virus concentration for two reasons: firstly, the SPR signal depends on the refractive index of the analyte and practically on the mass, which is high for viruses compared with individual proteins. Secondly, the virus particle presents the molecular target in multiple copies on its surface, which leads to enhanced binding to the immobilized aptamers. Furthermore the results show that neutravidin-binding does not affect the ability of the aptamer to bind VACV particles.

Finally, to prove the binding of A38 to VACV with both binding partners in solution, corresponding to *in vivo *conditions, Fluorescence Correlation Spectroscopy (FCS) was performed (Fig. 3c). This technique proved the binding of A38 to VACV particles by determination of the diffusion times for tetramethylrhodamine isothiocyanate (TMR)-labeled A38 and VACV alone and in combination. In detail, the respective diffusion times were 36 ± 2 μs for free TMR (443Da, black line), 165 ± 20 μs for TMR-labeled A38 (20 kDa, blue line), and 6.5 ± 0.9 ms for TMR-labeled VACV (350 nm in diameter, green line). When non-labeled VACV was added to a solution of TMR-labeled A38, the diffusion time of A38 increased to a magnitude similar to the one of TMR-labeled VACV (red line), which was attributable to the binding of A38 to VACV. Control experiments with CMV (brown line) and 200-nm latex beads (not shown) mixed with TMR-conjugated A38 showed diffusion times identical to those for TMR-labeled A38 alone, indicating no binding to both controls. These data prove that fluorescently labeled A38 binds specifically only to VACV particles, but not to control particles of similar size.

**Figure 4:**
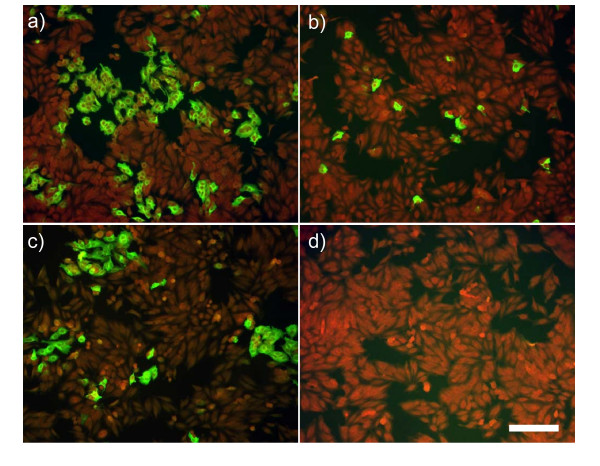
***In vitro *antiviral activity of A38. **Immunofluorescence microscopy of VACV-infected Hep2 cells incubated with A38 after 24 hours. (a) Cells infected with VACV at MOI of 0.1, (b) cells incubated with a mixture of VACV and 2.5 μM A38 or (c) VACV and 2.5 μM of the complete template library. (d) Control of non-infected cells. Bar, 100 μm. Red cells are counterstained with Evans's Blue.

To prove its biological activity, VACV infection of human Hep2 cells (Fig. 4), simian Vero E6 cells and primary chicken embryo fibroblast cells was determined in the presence of A38 by immunofluorescence assay (IFA). At a concentration of 2.5 μM, A38 clearly inhibited the spread of infection in all three cell types. When determining the cell viability under the influence of A38 at an equal concentration, it caused no increased cell death or apoptosis as proved by WST-1 cell proliferation assays (WST-1 Roche Applied Science, Germany) and annexin V/PI flow cytometric analysis (data not shown).

**Figure 5:**
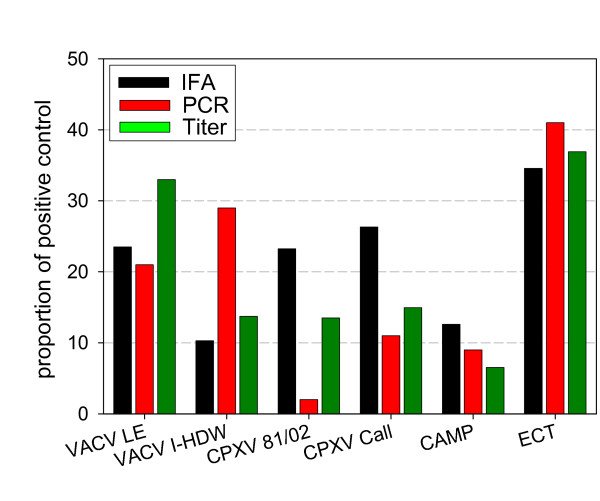
**Antiviral activity of A38 against various OPV**. OPV were propagated in cell culture in presence or absence of A38 as described above. The degree of infection in comparison to a positive control in the absence of A38 was determined by enumerating the number of infected cells (IFA, black bars), quantification of intracellular poxvirus DNA by real-time PCR (red bars) or determination of the poxvirus titer in the corresponding supernatant (green bars).

The antiviral potency of A38 was measured by an endpoint dilution (5 μM, 2.5 μM, 250 nM, 25 nM, 2.5 nM, 250 pM) and immuno-fluorescence-based assay in Hep2 cells against VACV (Fig. 4), followed by determining the ratio of infected/non-infected cells. A38 inhibited VACV entry with a 50% inhibitory concentration (IC_50_) value of 0.59 μM. The antiviral effect exerted by A38 was neither due to cytotoxicity nor to induced apoptosis. A38 had no significant effect on cell viability in cytotoxicity assays (WST-1, Roche, Germany). The compound concentration at which uninfected Hep2 cell proliferation was inhibited by 50% (CC_50_) was > 10 μM, which was above the available synthesized concentration for A38. This resulted in a selective index (SI) of > 16.95 (SI = CC_50_/IC_50_). A similar antiviral activity of A38 could be detected against a broad spectrum of OPV-like Cowpox virus, Ectromelia virus and Camelpox virus as shown by IFA, by quantitative real-time PCR of viral DNA used as described previously [26] and by virus titration of the corresponding supernatants (Fig. 5). Reduced virus proliferation could be confirmed in all experiments. Taken together, these results demonstrate that A38 binds to a target common to all OPV, is a potent and specific inhibitor of OPV. However, the inhibition of Ectromelia virus proliferation was less pronounced.

**Figure 6:**
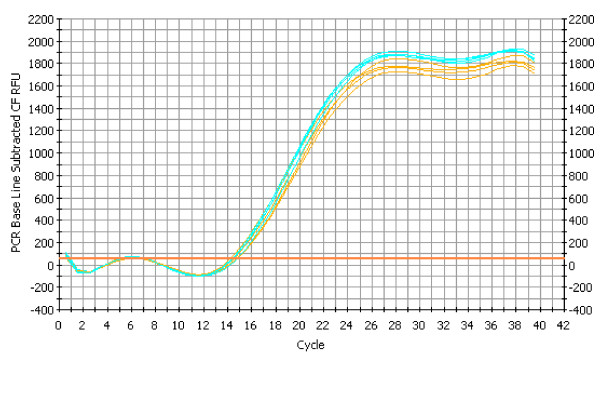
**Stability of DNA aptamer A38**. A38 was incubated for 24 h at 37°C in water or, in comparison, in serum. No significant shift of C_T _value was observed by quantitative real-time PCR for the serum-incubated aptamer (brown lines, n = 3) in comparison to the fresh aptamer A38 (blue lines, n = 2).

Since a potential therapeutic use of A38 in OPV infections requires sufficient nuclease resistance of the DNA aptamer, a precondition that unmodified RNA aptamers often fail to meet, its stability was evaluated by real-time PCR as described above. For that purpose, A38 was incubated in serum for 24 h at 37°C. As shown by the nearly identical C_T _values for A38 in water and in serum, no significant degradation of A38 was detected by quantitative PCR after a 24-h incubation in serum (Fig. 6). A38 still inhibited virus dissemination after 7 days in medium to a degree similar to fresh A38, as confirmed by IFA (data not shown).

## Discussion

When the first aptamers were developed 15 years ago, it was predicted that they had an enormous potential as a feasible alternative to antibodies. Although the application of antibodies for the detection and therapy of infectious agents is well established, aptamers have some potential advantages over antibodies that may fill the gaps that antibody-based applications possess and that expedite the use of aptamers in the virologist's laboratory [7]. These advantages include their small size, eased cell penetration, rapid and cheap synthesis including a variety of chemical modifications and the fact that aptamers are non-immunogenic. Probably their crucial benefit is a selection process performed *in vitro*. Thus, aptamers can be selected against targets that are either weakly immunogenic or toxic, like toxin proteins [27]. These targets are usually not applicable for *in vivo *production of monoclonal antibodies in mice; however, highly affine aptamers have been selected against some of these problematic targets [5]. Moreover, the chemical and physical conditions for aptamer selection can be adapted to the real environment in which the aptamer will finally be applied. This includes cross-selection against similar targets that have to be excluded from an aptamer's detection pattern.

With the FDA approval of the first aptamer-based drug Macugen and the recent publication of an aptamer-based cocaine sensor, aptamers are starting to tap their full potential with the increasing need for additional specific detection tools.

Most of the aptamers developed have been selected by SELEX or modifications of SELEX. In this study we evaluated a new selection procedure for aptamers. As demonstrated for the selection of OPV-specific DNA aptamers, the MonoLEX method is a straightforward procedure for the identification of high-affinity aptamers to a target structure of interest. Although SELEX has proven to generate specific aptamers for several targets, MonoLEX can be a valuable alternative for aptamer selection. Based on one affinity chromatography, an optimized enrichment of high-affinity aptamers is obtained by physical segmentation of the affinity resin.

The affinity of the bound aptamers is expected to decrease slightly in the direction of the outlet of the column. However, practical experience shows that the oligonucleotides assort in distinct clusters along the column. Column segmentation allows a direct desorption of high-affinity aptamers from individual column fragments, preventing their possible loss due to an amplification efficiency lower than that of low-affinity binders. The combination of differently coated affinity chromatography columns allows the depletion of aptamers that may also bind to structures closely related to the target, therefore increasing the specificity of the aptamers selected.

As shown by several analytical techniques, the selected DNA aptamer A38 possesses a considerable stability in binding to VACV. This binding could not only be demonstrated with immobilized virus particles on a dot blot, but also vice versa with immobilized aptamers as shown by Surface Plasmon Resonance Spectroscopy, a situation found on detection sensors for poxvirus particles. As described among others for HCV [28], A38 could be immobilized on sensor surfaces to identify poxvirus particles in complex matrices, as frequently seen in samples suspected to be of bioterrorist origin. The development of such a sensor has already been considered. Concerning the therapeutic application of A38, it was also important to prove its binding to OPV with both partners in solution. To this end, we performed FCS studies and demonstrated the binding of A38 to VACV particles, but not to CMV or latex beads of similar size. While FCS proved to be a reliable and sensitive method to demonstrate aptamer-virus interactions in solution, the binding of A38 to VACV-infected cells could not be observed by confocal fluorescence microscopy. The reason for this failure is still not clear but may be a problem of sensitivity. Although the binding partner of A38 on OPV particles is still unknown, it must be highly conserved and present on all different OPV. The *in vitro *replication of several OPV could significantly be inhibited in the presence of A38 in a concentration-dependent manner, with IC_50 _values of 0.59 μM, a concentration that is in agreement with previously published aptamers specific for other viruses [17]. Further studies have to evaluate the mechanism of inhibition and the potential to inhibit OPV replication in animal models.

## Conclusion

The need for specific diagnostic or therapeutic tools in infectious diseases is obvious. Here we present a new selection approach for aptamers, called MonoLEX, and describe the selection of an OPV-specific aptamer. According to MonoLEX, the amplification of aptamers from affinity-column segments after complete depletion of low-affinity binders by affinity chromatography might be a fast and reliable technology for isolation of highly affine aptamers. Especially in scenarios where infections with new, emerging pathogens have to be detected or treated, a fast technique providing specific detection tools may be helpful. The spectrum for different applications of aptamers like A38, either on a sensor for OPV detection or for treatment of infections, requires an extremely high stability of the aptamer. The use of DNA aptamers facilitates such a high stability without modifications, even in body fluids.

With the improving automation of the aptamer selection process and the growing knowledge of pathogen-specific targets, the number of aptamers used in diagnostics and therapy of infectious diseases will dramatically increase in the future.

## Methods

### Virus preparation

Vaccinia virus (VACV, strain NYCDH, ATCC #VR-1536), VACV (strain Lister Elstree), cowpoxvirus (CPXV, strain 81/02), mousepoxvirus (ECTV strain Nü-1) and camelpoxvirus (CMLV, strain CP19) were propagated in Hep2 cells (ATCC CCL-23) at a multiplicity of infection (MOI) of 0.25 following standard procedures with Dulbecco's Modified Eagle Medium (DMEM) containing 1 g/L D-glucose, L-glutamine and 25 mM HEPES buffer. Infected cells were incubated for approximately 4 days until a pronounced cytopathic effect was observed. Virus-containing supernatant and cells were harvested and subsequently separated by centrifugation (10 min at 1000 × *g*) after freeze thawing. Virus-containing supernatant was titered according to Reed and Münch [29] and stored at -75°C until use. For inactivation prior to selection, the virus suspension was heat-treated at 60°C for 1 h and checked for infectious activity by cell culture as described above. As control virus for binding experiments, CMV was kindly provided by Stefanie Thulke, Charité Berlin, Germany.

### Selection of high-affinity-binding aptamers by MonoLEX

A MonoLEX selection process comprised the following steps: Chemical coupling of the target (VACV) to the affinity column, incubation with the combinatory library, extensive washing to elute non-binding oligonucleotides, physical segmentation of the affinity column, PCR amplification of the aptamers bound to different column fragments and finally an aptamer dot blot assay to evaluate the binding specificity.

The selection experiments started from a combinatory library that was 64 bases long (5'-TACGACTCACTATAGGGATCC-N_7_-A-N_7_-A-N_6_-GAATTCCCTTTAGTGAGGGTT-3') including 20 random nucleotide positions and two terminal PCR primer sequences diluted in DMEM. For selection, an affinity capillary column was coated with heat-inactivated VACV (strain NYCDH) particles. To reduce unspecific binding, the complete template library was first applied to an affinity capillary column covalently coated with the cell culture supernatant of non-infected Hep2 cells. After application of the remaining combinatory library to the selection column, low-binding oligonucleotides were washed off with approximately the 5000-fold column volume of Tris buffer (pH 7.3, 0.1% TWEEN-20), DMEM (0.1% TWEEN-20) and phosphate buffer (pH 7.5, 0.1% TWEEN-20). The column was then cut into slices. Target-bound aptamers were desorbed from the column slices by denaturation of the target and amplified by quantitative real-time PCR on an iCycler (BioRad, Munich, Germany), using 2 pmol of the primers AP7 TACgACTCACTATAgggATCC and AP3: AACCCTCACTAAAgggAATT (Metabion, Martinsried, Germany) in a SybrGreen Mastermix (Cambrex, NJ, USA). The mixture of such amplified aptamers is called "polyclonal aptamers". Cycling conditions were as follows: Initial denaturation at 95°C for 5 min and 40 repeats of denaturation at 95°C for 15s, primer annealing at 50°C for 30s and elongation at 68°C for 30s. Subsequently to PCR, a fluorescence curve melting analysis (FCMA) was performed, cooling down the PCR reaction product to 50°C and increasing the temperature with a maximum ramping rate up to 90°C. Fluorescence was monitored continuously and melting peaks were calculating with the iCycler software.

### Pyrosequencing

Pyrosequencing was performed with a PyroMark ID System (Biotage, Sweden) using the Pyro Gold kit. Briefly, aptamer candidates were amplified by PCR as described above, applying one biotinylated primer AP3. Single-stranded DNA was prepared by denaturation in NaOH and binding to sepharose beads as recommended by Biotage. The single-stranded DNA was sequenced with primer AP7 in the sequencing mode of the PyroMark ID System. Usually, the complete aptamer sequence could be determined unambiguously.

### Aptamer synthesis

Aptamer A38 was purchased as lyophilized oligonucleotide (Oligoservice, Berlin, Germany), either non-modified, 5' biotinylated or 5' tetramethylrhodamine isothiocyanate (TMR) labeled. All aptamers were reconstituted in Tris-HCL (10 mM, pH 8.4) as 100 μM stock solutions and stored at -20°C until use for further experiments.

### Aptamer blotting assay

To validate the presence of target specific aptamers in the aptamers derived from the individual slices of the affinity column, a PCR-enhanced dot blot assay was performed. Briefly, the target VACV particles were chemically immobilized to a polypropylen tube. Polyclonal aptamers were purified by agarose gel electrophoresis from PCR mastermix components and incubated in the tubes overnight. After ample washing the immobilized aptamers were amplified by quantitative PCR as described above.

### Dot blot

A38 was tested at various concentrations against VACV, non-infective cell culture supernatant and CMV. 30 μL of sucrose-cushion-purified VACV (~10^7 ^particles) were spotted onto a 0.2 μm PVDF membrane (Schleicher & Schuell, Dassel, Germany). After incubation with blocking buffer (PBS, 0.1% Tween 20 und 10% low-fat milk powder), the membrane was incubated with varying amounts of biotin-conjugated A38 (0.01–10 nM) for 1 h at RT. Following repeated washing with PBS and incubation with a streptavidin peroxidase conjugate (Dianova, Hamburg, Germany) for 1 h, the detection was performed by chemiluminescence using SuperSignal^® ^West Pico (Pierce, USA) according to the manufacturer's instructions.

### Fluorescence Correlation Spectroscopy (FCS)

The binding of the aptamer to VACV was assayed by Fluorescence Correlation Spectroscopy (FCS)[30]. We used a confocal microscope ConfoCor (Carl Zeiss, Jena; Evotec Biosystems, Hamburg, Germany) and analyzed the diffusion of dye-labeled monomers and complexes by the normalized autocorrelation function (ACF), as described previously [31]. The fluorescence fluctuations of the marker dye, tetramethylrhodamine isothiocyanate (TMR), were recorded for 5 min at RT and submitted to autocorrelation analysis using the hardware and software by Evotec Biosystems (Hamburg, Germany). The autocorrelation function was analyzed in terms of particle diffusion through the confocal volume and the intrinsic triplet state dynamics of the fluorophor TMR [32]. The analysis yielded the number of particles and the mean dwell time (diffusion time) of each particle species in the confocal volume. Total particle numbers between 0.5 and 1.5 were observed. Data were rescaled to a total particle number of one for better comparison. The calculated diffusion times were characteristic for TMR, A38-TMR and VACV-TMR. Saturation of the binding of TMR-labeled A38 to non-labeled VACV was indicated by a complete shift of the autocorrelation function (ACF) from a shorter to a longer correlation time characteristic for VACV-TMR. The following concentrations were applied: 3 nM TMR, 5 nM A38-TMR, 10^8 ^PFU/ml VACV-TMR, 10^8 ^PFU/ml non-labeled VACV and 10^8 ^PFU/ml non-labeled CMV in PBS buffer.

### Surface Plasmon Resonance measurements (Biacore)

Binding experiments were done with the SPR-based instrument Biacore™2000 (Biacore, Freiburg, Germany) and sensor chips CM4, using the control software version 2.1 and evaluation software version 3.0 (Biacore AB, Uppsala, Sweden). The running buffer was PBS containing 0.005 % (v/v) Tween 20. This buffer was also used for sample dilution. The temperature of the flowcells was 25°C.

For the immobilization of Neutravidin, the carboxymethyl dextrane matrix of the chip was activated for 7 min with 35 μl of a mixture of 0.2 M 1-ethyl-3-(3-dimethylaminopropyl)carbodiimide hydrochloride (EDC) and 0.05 M N-hydroxysuccinimide (NHS). Neutravidin (Pierce, Rockford, USA) was diluted to 20 μg/ml in 10 mM phosphate pH 5.5 and injected into the activated flowcell 2 for 5 minutes. Non-reacted sites were blocked with 1 M ethanolamine pH 8 for 1 min. The amount of immobilized Neutravidin was 2100 resonance units (RU) for flowcell 2. The control flowcell 1 was only activated and blocked.

Binding experiments were performed with aptamer A38 that was injected into flowcell 2 at a concentration of 1 μM in PBS for 5 min, yielding 480 RU for bound aptamer. After 5 min of washing with buffer, the dissociation of aptamer was constantly slow, and the virus suspension (10^9 ^particles/ml) was injected into flowcells 1 and 2 for 5 min. Bound particles were then eluted with 50 mM sodium carbonate for 1 min. Finally, a tenfold diluted virus suspension was injected (Fig. 3b, left panel). Specific binding sensorgrams were obtained by normalization to the curve of the control flowcell 1. The background was set to zero, and the injection start was synchronized (Fig 3b, right panel). Kinetic evaluation of the binding was not possible, as the binding of virus particles is a multisite attachment to the aptamer layer.

### Immunofluorescence Assay (IFA)

5 × 10^3 ^Hep2 cells were propagated in 96-well plates (Nunc, Wiesbaden, Germany) for 24 h at 37°C. Cells were either infected with VACV at a MOI of 0.1 alone or together with 2.5 μM A38 or 2.5 μM of the combinatory library. Cells were incubated for another 24 h at 37°C. For visualization OPV-infected cells were fixed in 4% formalin and stained with a polyclonal human anti-pox IgG (1:500, Omrigam, USA), followed by a FITC-conjugated goat anti-human IgG (1:50, Caltag Laboratories, USA) according to standard procedures and contrasted by Evans Blue staining. The number of infected cells was determined by fluorescence microscopy. Briefly, four representative pictures ((??)) were taken. The total cell number, stained with Evans Blue, and the number of infected cells, stained with the anti-poxvirus serum, were enumerated by application of the software package ImageJ .

## Authors' contributions

AN designed and coordinated the study and drafted the manuscript. AKu performed cell culture, participated in FCS, Dot Blot and IFA work and drafted the manuscript. AD and DM performed cell culture, Dot Blot and IFA work. OP and WJ initiated work with FCS and helped to draft the manuscript. HS performed FCS work. FS and WS initiated and performed work with Biacore. AKa helped to design the study, performed aptamers blotting assay and helped to draft the manuscript. CD performed the aptamers selection. GP participated in the study design and coordination.

## References

[B1] Jahrling PB, Fritz EA, Hensley LE (2005). Countermeasures to the bioterrorist threat of smallpox. Curr Mol Med.

[B2] Yang G, Pevear DC, Davies MH, Collett MS, Bailey T, Rippen S, Barone L, Burns C, Rhodes G, Tohan S, Huggins JW, Baker RO, Buller RL, Touchette E, Waller K, Schriewer J, Neyts J, DeClercq E, Jones K, Hruby D, Jordan R (2005). An orally bioavailable antipoxvirus compound (ST-246) inhibits extracellular virus formation and protects mice from lethal orthopoxvirus Challenge. J Virol.

[B3] Zhang Z, Blank M, Schluesener HJ (2004). Nucleic acid aptamers in human viral disease. Arch Immunol Ther Exp (Warsz ).

[B4] Breaker RR (2004). Natural and engineered nucleic acids as tools to explore biology. Nature.

[B5] Hesselberth JR, Miller D, Robertus J, Ellington AD (2000). In vitro selection of RNA molecules that inhibit the activity of ricin A-chain. J Biol Chem.

[B6] Ulrich H, Magdesian MH, Alves MJ, Colli W (2002). In vitro selection of RNA aptamers that bind to cell adhesion receptors of Trypanosoma cruzi and inhibit cell invasion. J Biol Chem.

[B7] James W (2007). Aptamers in the virologists' toolkit. J Gen Virol.

[B8] Nolte A, Klussmann S, Bald R, Erdmann VA, Furste JP (1996). Mirror-design of L-oligonucleotide ligands binding to L-arginine. Nat Biotechnol.

[B9] Schmidt KS, Borkowski S, Kurreck J, Stephens AW, Bald R, Hecht M, Friebe M, Dinkelborg L, Erdmann VA (2004). Application of locked nucleic acids to improve aptamer in vivo stability and targeting function. Nucleic Acids Res.

[B10] Tuerk C, Gold L (1990). Systematic evolution of ligands by exponential enrichment: RNA ligands to bacteriophage T4 DNA polymerase. Science.

[B11] Ellington AD, Szostak JW (1990). In vitro selection of RNA molecules that bind specific ligands. Nature.

[B12] Ellington AD (1994). RNA selection. Aptamers achieve the desired recognition. Curr Biol.

[B13] Wilson DS, Szostak JW (1999). In vitro selection of functional nucleic acids. Annu Rev Biochem.

[B14] Hermann T, Patel DJ (2000). Adaptive recognition by nucleic acid aptamers. Science.

[B15] Chen F, Zhou J, Luo F, Mohammed AB, Zhang XL (2007). Aptamer from whole-bacterium SELEX as new therapeutic reagent against virulent Mycobacterium tuberculosis. Biochem Biophys Res Commun.

[B16] Dey AK, Griffiths C, Lea SM, James W (2005). Structural characterization of an anti-gp120 RNA aptamer that neutralizes R5 strains of HIV-1. RNA.

[B17] Wang J, Jiang H, Liu F (2000). In vitro selection of novel RNA ligands that bind human cytomegalovirus and block viral infection. RNA.

[B18] Jeon SH, Kayhan B, Ben-Yedidia T, Arnon R (2004). A DNA aptamer prevents influenza infection by blocking the receptor binding region of the viral hemagglutinin. J Biol Chem.

[B19] Gopinath SC, Misono TS, Kawasaki K, Mizuno T, Imai M, Odagiri T, Kumar PK (2006). An RNA aptamer that distinguishes between closely related human influenza viruses and inhibits haemagglutinin-mediated membrane fusion. J Gen Virol.

[B20] Thiel K (2004). Oligo oligarchy-the surprisingly small world of aptamers. Nat Biotechnol.

[B21] Nimjee SM, Rusconi CP, Sullenger BA (2005). Aptamers: an emerging class of therapeutics. Annu Rev Med.

[B22] Stojanovic MN, Landry DW (2002). Aptamer-based colorimetric probe for cocaine. J Am Chem Soc.

[B23] Joyce GF, Cech TR (1989). Building the RNA world: evolution of catalytic RNA in the laboratory. Molecular Biology of RNA.

[B24] Yarus M (2006). RNA oligonucleotides specifically binding to guanine. Molecular Biology of RNA.

[B25] (2007). M-fold software. http://www.bioinfo.rpi.edu/applications/mfold.

[B26] Nitsche A, Stern D, Ellerbrok H, Pauli G (2006). Detection of Infectious Poxvirus Particles. Emerg Infect Dis.

[B27] Tang J, Yu T, Guo L, Xie J, Shao N, He Z (2007). In vitro selection of DNA aptamer against abrin toxin and aptamer-based abrin direct detection. Biosens Bioelectron.

[B28] Lee S, Kim YS, Jo M, Jin M, Lee DK, Kim S (2007). Chip-based detection of hepatitis C virus using RNA aptamers that specifically bind to HCV core antigen. Biochem Biophys Res Commun.

[B29] Reed LJ, Münch H (1938). A simple method of estimating fifty per cent endpoints. Am J Hyg.

[B30] Widengren J, Mets, Zander C, Enderlein J and Keller RA (2002). Conceptual basis of Fluorescence Correlation Spectroscopy and related techniques as tools in bioscience. Single-Molecule Detection in Solution - Methods and Applications.

[B31] Hasler K, Panke O, Junge W (1999). On the stator of rotary ATP synthase: the binding strength of subunit delta to (alpha beta)3 as determined by fluorescence correlation spectroscopy. Biochemistry.

[B32] Widengren J, Mets, Rigler R (1995). Fluorescence Correlation Spectroscopy of Triplet States in Solution: A Theoretical and Experimental Study. J Phys Chem.

